# Ten Simple Rules for Choosing between Industry and Academia

**DOI:** 10.1371/journal.pcbi.1000388

**Published:** 2009-06-26

**Authors:** David B. Searls

One of the most significant decisions we face as scientists comes at the end of our formal education. Choosing between industry and academia is easy for some, incredibly fraught for others. The author has made two complete cycles between these career destinations, including on the one hand 16 years in academia, as grad student (twice, in biology and in computer science), post-doc, and faculty, and on the other hand 19 years in two different industries (computer and pharmaceutical). The following rules reflect that experience, and my own opinions.

## Rule 1: Assess Your Qualifications

If you are a freshly minted Ph.D., you know that you will need a good post-doc or two before you can be seriously considered for a junior faculty position. If you're impatient, you might be thinking of industry as a way to short-circuit that long haul. You should be aware that companies will strongly consider your post-doctoral experience (or lack thereof) in determining your starting position and salary. While you may not relish extending your indentured servitude in academia, any disadvantage, financial and otherwise, can quickly be made up in the early years of your career in industry. In other words, trying to get off the mark quickly is not necessarily a good reason to choose industry over academia.

On the other hand, you may have completed an undergraduate or Master's program with a view to going to industry all along, with never a thought of an academic career. You should still consider the point of the previous paragraph. While abbreviated “practical” bioinformatics training programs can be excellent, a Ph.D. is a significant advantage in all but the most IT-oriented positions in industry, at least at the outset. This is not to discourage anyone from embarking on a fast-track-to-industry program if their heart is in it, but be aware that the further you climb the educational ladder, the higher and faster you can start when you step across to the business ladder, and the better you will compete for a job in the first place. The days are long past when bioinformaticists were in such short supply that any qualification would do.

If you are an old hand and have already notched up a post-doc or two, take stock of your star power. This unspoken but universally understood metric encompasses such factors as whom you've trained with, where you've published (and how much), and what recent results of yours are on everyone's lips. If you are fortunate enough to have significant capital in this department, then the world may be your oyster, but you still need to consider where you will get the greatest leverage. While your stardom may be less taken for granted in industry, my feeling is that academia is a better near-term choice in such circumstances. Consider that it was in academia that you achieved the success you own thus far, so you obviously “get it.” The simple fact is that academia is rather more of a star system (as in Hollywood) than is industry.

Finally, if you count among your qualifications a stint in industry already, as an intern or perhaps as part of a collaboration, you will not only be in a better position to compete for a permanent job, but you will be much better prepared to make the decision facing you. Stated another way, if you are seriously considering industry as a career path, you should probably have already taken advantage of the many opportunities out there to dip your toes in the water.

## Rule 2: Assess Your Needs

In taking stock of your needs, and perhaps those of your family, a decent living is generally at or near the top of the list. Salaries are still higher in industry, though the gap is not nearly so wide as it once was. If you need a quick infusion of cash, companies may offer signing bonuses, though again these were more common when bioinformatics was a rarer commodity.

Industry offers forms of compensation unavailable in academia, and you will need to consider how to value them relative to your present and future needs. Despite recent bad press, bonus systems are often part of the equation, and depending on your entry point they may constitute a significant percentage of total compensation. There is a tendency among academics to discount bonus programs in their comparison shopping, sometimes to zero, and this is a mistake. Bonuses are considered core aspects of compensation in most companies, and though they always have a performance-based multiplier, the base levels have historically been fairly dependable. That said, these are tough times in industry, and there are no guarantees. Your best strategy is to understand the reward system thoroughly, ask for historical data, and avoid comparing only base salaries unless you are extraordinarily risk-averse.

Share options are another matter. While in the past these were very attractive, and fruitful in practice, most industry types will tell you frankly that any options they've received in the past decade are deep underwater and a deep disappointment. Many consider pharma shares (and therefore options) to be a bargain at the moment, but that's between you and your financial adviser to assess. In any case, it is not a short-term consideration, since options typically take several years to vest.

If you are looking at biotech, however, share options and similar ownership schemes need to be a key consideration, since these are a major rationale for assuming risk—more on that below.

Finally, you may have more specific needs to consider, such as a spouse also in need of a job. The two-body problem has always been tougher in academia than in industry, and probably always will be. If you are both academics, note that industry often has good contacts with local universities, and can facilitate interviews. Being a star certainly helps, so don't be afraid to negotiate. In fact, a general rule of thumb is that it never hurts to make your specific needs known, within reason. Academia will try to accommodate them as a community, while on the other hand business (particularly large, diversified companies) may have resources to address them that you wouldn't have expected. Nobody wants to hear a peremptory demand, but if a company wants you, be sure to let them know anything that might offer them a way to attract you.

## Rule 3: Assess Your Desires

There are needs, and then there are desires. Do you want riches? Fame? A life at the frontiers of knowledge? The hurly-burly of the business world? How do you really feel about teaching, publishing, managing, interacting, traveling, negotiating, collaborating, presenting, reporting, reviewing, fundraising, deal-making, and on and on? Though it may seem obvious, this is a good time to decide what really drives you.

First, the obvious. Do you want to teach? If lecturing is in your blood, your decision is made, although if a smattering will suffice you may have the option from within industry of an adjunct academic appointment. (By the same token, if you are not so enchanted with lecturing, grading, tutoring, etc., there are often options for research track professorships that minimize teaching duties.) Do you want to publish? While it will always be “publish or perish” in academia, it is certainly possible to grow your CV in industry, and it can even enhance your career, depending on the company. However, it might be largely on your own time, and you will likely encounter restrictions in proprietary matters, though in practice you can generally find ways to work within them. Ask about publication at the interview, both policies and attitudes, and watch out for any defensiveness.

An important question, surprisingly often overlooked, is how you want to actually spend your time, day by day and hour by hour. In academia, you will immediately be plunged into hands-on science, and your drivers will be to start out on your career by getting results, publishing, networking, and building your reputation with a view to impressing your tenure committee. A career in industry may put more of an early emphasis on your organizational aptitude, people skills, powers of persuasion, ability to strategize and execute to plan, etc.; in terms of growing your reputation, your audience will be the rather narrower community of your immediate management. A somewhat more cynical view would be that in business you will spend seemingly endless hours in meetings and writing plans and reports, while in academia you will spend all that time and more in grantsmanship—in this regard, you must pick your poison.

Finally there is the elephant-in-the-room question: Do you want to make money, or to help people? This is, of course, a false dichotomy, but many people consciously or unconsciously frame the decision in just this way, and you had best deal with it. Try thinking of it not so much in terms of the profit motives of the respective institutions, but in terms of the people with whom you would spend your career. You should have encountered a good sampling of scientists from industry during meetings, internships, collaborations, interviews, etc. (or in any case you should certainly try to do so before making judgments). If you are left in any doubt as to their ethics or sincere desire to relieve human suffering as efficiently as possible, or if you feel these are somehow trumped by the corporate milieu, then by all means choose academia—but only after applying analogous tests to the academics you already know well. In my experience, business doesn't have a monopoly on greed, nor are humanitarian impulses restricted to academia. That said, in the final analysis you must be comfortable with your role in the social order and not finesse the question.

## Rule 4: Assess Your Personality

Not surprisingly, some personality types are better-suited to one environment or the other. Raw ambition can be viewed as unseemly in either case, but there is more latitude for it in industry, and greater likelihood of being recognized and rewarded sooner if you are “on the go.” In fact, one of the clearest differences between academia and industry are their respective time constants. Although the pace of academia may have quickened of late, it is still stately by comparison with industry, and much more scheduled (so many years to tenure, so many months to a funding decision, etc.). If you are impatient, industry offers relatively fast-paced decision-making and constant change. If you thrive more under structured expectations, academia would be better for you, for although industry has all the trappings of long-range strategies and career planning, the highly reactive environment means these are more honored in the breach. For one thing, reorganizations are common, and in the extreme case mergers (I have experienced two) can reset everything, for good or ill, and devour many months.

This is not to say that all is chaos—industry certainly favors a goal-directed personality, but with plenty of flexibility. On the other hand, flexibility is more the hallmark of academic research, where you will have the opportunity to follow wherever the science leads, once you are running your own shop. In industry, the flexibility is more of the conforming sort, since you won't be able to investigate every promising lead and change your research direction at will. In academia, diverging from the Specific Aims of a grant may be a problem when the time comes to renew, but the risk is yours, as is the reward. In industry, you can make the case for a new program of research, but the decision is management's and will be guided by business considerations. The “lone wolf” or “one-person band” may be increasingly rare in academia in an age of collaboration, but it is unheard of in industry, where being able to work in teams with specialized division of labor is essential. It should be apparent, as well, that mavericks and quirky personalities tend to do better in academia.

The pecking order in industry is deeper and more pyramidal than in academia, and you might end up languishing in a pay grade (or feel like you are), but there are usually plenty of opportunities for lateral moves and a variety of experiences—not to mention that it's easier to switch companies than colleges. In industry, one does need to be able to thrive in a hierarchy; you will always answer to someone, though the degree to which you are monitored will vary. By the same token, if your personality is such that climbing a management ladder and assuming steadily greater responsibility suits you, industry is built for that, and plenty of management training is on offer in larger companies. Learning to manage is much more hit-or-miss in academia; opportunities to lead large organizations are rare (and to manage them actively rather than by consensus, rarer still).

If your personality type is that of a risk-taker, biotechs and/or startups may fit you to a tee. These are the wild and wooly end of the industry spectrum, and the risks and rewards are well-known. You will work longer hours than in large pharma, and maybe even more than in academia. You will most likely share more in ownership, and learn entrepreneurial skills that will serve you well, once the bug has bitten. Bear in mind the very common pattern of faculty spinning off startups or otherwise participating in boards and the like, not to mention staking out intellectual property (shared with their university); thus, you may well be able to scratch this itch from the vantage of academia as well.

A final word about politics. Whether you are an enthusiastically political animal, or abhor this aspect of the human condition, you will encounter plenty of politics in both academia and industry. The flavors differ, to be sure. As a student you doubtless heard the clichés about tedious academic committees and underhanded deans, but you have probably had more exposure to the realities behind those stories than the corresponding ones about the dog-eat-dog corporate world. Company politics, I would hazard to say, are more transparent—the maneuvering more open and the motives more apparent. The results are often more life-altering, unbuffered by tenure and academic convention. Again, it is a matter of taste, but in my opinion the differences are overblown, for the simple reason that people are the same everywhere, in both environments governed by an underlying sense of fair play, but also occasional opportunism.

## Rule 5: Consider the Alternatives

As I've suggested, the choice you face is far more fine-grained than simply that between industry and academia. Industry is a spectrum, from large pharma to mature biotech to startup. By the same token, the academic side has at one extreme the research powerhouses, where you will be judged by volume of grants, and at the other the teaching institutions, which may not even have graduate departments. Unless you are very sure of yourself, you'd be well-advised to consider the full range, given the competition you may face.

Also, don't neglect other careers that may value your training. If you love the language, consider science journalism, either writing or editing—*Science* and *Nature* have large staffs, and you will often encounter them and representatives of other journals at the same scientific meetings you attend. The same is true of government agencies such as the NIH, NSA, DOE, and so forth, where grants administration is very actively tied to research trends and can be an entrée into the world of science policy. There are many more such positions when foundations, interest groups, and other private funding bodies are included. If you have a knack for business, many management consulting firms have scientific and technical consulting arms that value Ph.D.s and offer intensive training opportunities, and, though it may not be attractive at the moment, a career as a financial analyst specializing in biotech is yet another possibility.

## Rule 6: Consider the Timing

The current business environment cannot help but be among your considerations. Pharma has certainly been contributing to the unemployment rolls of late. Corporate strategies, which used to be very similar across the sector, have started to diverge, so that some companies are divesting bioinformatics at the same time that others are hiring computational types disproportionately as they place more of an emphasis on mathematical modeling, systems approaches, pharmacogenomics, drug repurposing, and the like. Overall, though, the industry trend has been to shrink R&D, and this may well continue through a round of consolidation, with several mega-mergers now under way. As noted above, mergers are times of upheaval, carrying both risk and opportunity, and usually a period in limbo as well. At the same time, it is worth bearing in mind that a corollary of downsizing is outsourcing, so that there may be new opportunities for startups and even individual consultants.

For much of the last decade, academia has also been in the doldrums, as NIH budgets have effectively contracted. As I write this, things are definitely looking up, with prospects for renewed funding of science and even near-term benefits to the NIH and NSA from the Obama stimulus package. Whether universities will respond proportionately with faculty hiring, given the losses in their endowment funds and cutbacks in salaries and discretionary spending, remains to be seen. There is a lot of slack to be taken up, and in particular a backlog of meritorious grant applications that are now being reconsidered. Nevertheless, on balance, an academic career has to be somewhat more promising today than a year ago, and a career in pharma rather less so, in the opinion of the author.

## Rule 7: Plan for the Long Term

Having noted the current situation in Rule 6, it's important also to say that a career decision should be made with the long haul in mind. The business cycle will eventually reverse itself, and while the business model may need to change irrevocably, the aging population alone dictates that healthcare will be an increasing global priority. Likewise, history shows that growth in government funding for science waxes and wanes, with a time constant somewhat longer than a decade. Trying to optimize a career decision based on current conditions is a bit like trying to time the stock market—you are sure to be overtaken by events.

One approach is to choose some reasonably long time frame, perhaps a decade, and ask yourself whether you'd be content to have lived through the average ups and downs you'd experience in a given job over that period. In academia, that would include a tenure decision (rate your chances), a lot of grant applications with mixed success at best, and maybe some great students and really significant scientific contributions. In pharma or large biotech, it would encompass a couple of promotions, your own group and maybe a department, at least one merger or other big disruption, and several rounds of layoffs. In small business, it might include a failed startup (or two, or three), an IPO if you're lucky, and a lucrative exit strategy or long-term growth if you're really lucky.

If you game these scenarios with various probabilities, and use your imagination, it just might become clear which ones you have no stomach for, and which ones really hold your interest.

## Rule 8: Keep Your Options Open

Job-hopping is much more prevalent now than in days of yore, and you should consider this in your scenarios. In industry, there is little stigma attached to changing employers, and if you can tolerate the relocation and/or want to see the world, it is a more or less standard way to advance your career by larger-than-usual increments. This stratagem is far from unknown in academia, but perhaps a bit trickier to execute, though of course it is de rigueur if you fail to get tenure.

Of greater interest is the question of moving between academia and industry. From the former to the latter is fairly easy, but the reverse is not as common, for a variety of reasons. Superstar academics in relevant areas are in great demand in industry, to which they are often exposed through consulting or scientific advisory boards. There are multiple examples of senior academics taking over major R&D organizations in industry, sometimes orders of magnitude larger than anything they managed in academia, and you might even consider this well-trod path as a career goal from the outset.

It is not impossible to return to academia from industry, particularly if you were already quite prominent when you left, but if you start your career in industry you may be at a disadvantage unless you go to great lengths to maintain an academic-style publication record and CV. Important exceptions would be if the work that you did in industry was particularly novel and/or high-profile, or if your business experience is valued in the post you seek. Examples of the latter might be faculty positions with a prominent management component (centers, institutes, core facilities, and the like), or an interface role back to industry, or perhaps a joint business school appointment.

## Rule 9: Be Analytic

Approach the decision with the analytic skills you've learned to apply to scientific questions. Gather data from all available sources and organize it systematically. When you interview, don't just impress, but get impressions; record everything down to your gut feelings. Do some bibliometric or even social network analyses of your potential colleagues. Check the industry newsletters and blogs, albeit with a grain of salt, to get a sense of the mood around R&D units (not to be confused with manufacturing, sales and marketing, or other divisions, which may have completely different cultures within the same company).

You might even try out some decision theoretic methodologies, such as decision matrices and Bayesian decision trees, or run simulations on the scenarios of Rule 7. I recommend taking a look at expected utility theory and prospect theory, for an interesting quantitative excursion. But honestly, these suggestions are just a more sophisticated informatics version of the classic advice to “make a list of pros and cons,” which always makes one feel a little more in control.

## Rule 10: Be Honest with Yourself

Another homily: Now, if ever, is the time to be honest with yourself. Take a hard look at your qualifications, with as much objectivity as you can muster, and use these rules to decide where you would be best-suited and positioned for success. But even more importantly, deal with your emotional responses to industry and academia. If something is nagging at you, tease it out into the open, and try to decide if it is well-founded or not; if you can't decide, then you have to acknowledge it, and realize that it may not go away in the future either.

Finally, try to keep some perspective. Your career choice is important, but not irrevocable, and there are more consequential things in life. Don't let the decision process ruin what should be an exciting time for you.

